# UVA-1 therapy for sclerosing skin disease: A pilot study of patient and clinician-reported outcomes in a single-arm nonrandomized trial

**DOI:** 10.1016/j.jdin.2026.06.009

**Published:** 2026-06-23

**Authors:** Christiaan H. Noot, Zachary Hopkins, Mason M. Seely, Rachel M. Seifert, Laurel Gray, Vikram Sahni, Amber Jimenez, Aaron Secrest, Christopher B. Hansen

**Affiliations:** a Department of Dermatology, University of Utah Spencer F. Eccles School of Medicine, Autoimmune Skin Diseases Clinic, Salt Lake City, Utah; b Department of Dermatology, Christchurch Hospital, Te Whatu Ora-Waitaha Canterbury, Christchurch, New Zealand

**Keywords:** autoimmune, epidemiology, graft-vs-host disease, GVHD, limited scleroderma, medical dermatology, morphea, patient-reported outcomes, scleroderma, UVA-1

*To the Editor:* Sclerosing skin diseases (SSDs) are inflammatory connective tissue diseases that cause fibrosis and thickening of the skin. SSDs are challenging to treat and detrimentally impact quality of life (QoL).^1^^,^^2^ Common treatments include topicals (corticosteroids, calcineurin inhibitors, vitamin D), intralesional corticosteroids, and systemic immunosuppressives.^3^ Phototherapy has been proposed for SSDs, but data are limited. We explored the impact of UVA-1 therapy on patient-reported outcomes (PROs) and clinician-reported outcomes (CROs) in patients with SSDs.

Seventeen adult patients with SDD completed 30 sessions of UVA-1 therapy (50-90 J/cm^2^ per treatment) within a 100-day period at the University of Utah (average 2-3 sessions/week) (Supplementary Fig 1, available via Mendeley at https://data.mendeley.com/datasets/c9r3gjvksy/1). CROs and PROs were gathered at baseline and after 30 treatments by a single reviewer. SSD diagnoses included: morphea (*n* = 8), limited or diffuse cutaneous systemic sclerosis (SSc) (*n* = 8), and sclerotic chronic cutaneous graft versus host disease (GVHD) (*n* = 1). 15 (88.2%) were female, and the mean age (SD) was 47.2 (±16.5) years. No concomitant treatments were discontinued for participants and treatments included mycophenolate mofetil, hydroxychloroquine, prednisone, rituximab, and topical steroids. Although antibody profiles are linked with specific clinical manifestations and prognoses, treatment outcomes stratified by antibody profiles were not analyzed due to the small cohort size.^4^ See Supplementary, available via Mendeley at https://data.mendeley.com/datasets/c9r3gjvksy/1 for detailed demographic, antibody profile, and concomitant treatment data.

After 30 treatments, HAQ-DI (β = 0.01 [−0.07, 0.1]) and HAQ-DI pain scale scores (β = −10.9 [−22.1, 0.33]) showed non-significant improvement (*P* = .06). Skindex-16 scores improved in the emotional, symptoms, and functioning domains (β = −16.2 [95% CI −27.97, −4.51], β = −28.6 [−40.4, −16.93], and β = −9.1 [−17.8, −0.32] respectively). PROMIS-PF scores also demonstrated a small improvement (β = 2.12 [0.04, 4.2]) (Table I, Supplementary Table V, available via Mendeley at https://data.mendeley.com/datasets/c9r3gjvksy/1, disease-specific data).

**Table I:** PRO results

Questionnaire	Average baseline (SD)	Average treatment 30 (SD)	Coefficient (95% CI)	*P*-value
Skindex-16				
Emotional	60.35 (±20.29)	44.12 (±29.35)	−16.2 (−27.97, −4.51)	.007
Symptoms	56.18 (±24.63)	27.53 (±18.04)	−28.6 (−40.4, −16.93)	<.001
Functional	36.94 (±31.40)	27.88 (±24.61)	−9.1 (−17.8, −0.32)	.04
PROMIS-PF	44.19 (±9.95)	46.31 (±9.98)	2.12 (0.04, 4.2)	.05
HAQ				
Disability Index	2.22 (±0.37)	2.24 (±0.36)	0.01 (−0.07, 0.1)	.73
Pain Scale	42.65 (±29.01)	31.76 (±24.08)	−10.9 (−22.1, 0.33)	.06

*HAQ*, Health Assessment Questionnaire; *PRO*, patient-reported outcome; *PROMIS-PF*, Patient Reported Outcome Measurement Information System-Physical Functioning.

Similar improvements were seen in CROs, including the mRSS (β = −7.5 [−11.2, −3.82]), LoSAI (β = −17.9 [−24.5, −11.3]), PGA Activity scores (β = −35.1 [−47.1, −23.1]), and HAMIS scoring system (β = −3.25 [−4.6, −1.9]). We did not find evidence for improvement in PGA Damage scores (β = 6.25 [−4.38, 16.9]) and LoSDI (β = −0.13 [−5.42, 5.17]) (Table II).

**Table II:** CRO results

Clinical assessment	Average baseline (SD)	Average treatment 30 (SD)	Coefficient (95% CI)	*P* value
mRSS (only SSc)	21.75 (±12.76)	14.25 (±9.02)	−7.5 (−11.2, −3.82)	<.001
LoSAI (only morphea)	21.38 (±14.22)	3.5 (±3.78)	−17.9 (−24.5, −11.3)	<.001
LoSDI (only morphea)	22.5 (±13.78)	22.38 (±17.38)	−0.13 (−5.42, 5.17)	.96
HAMIS (only SSc)				
Left Hand	12.63 (±6.65)	9.38 (±6.86)	−3.25 (−4.6, −1.9)	<.001
Right Hand	13.75 (±5.28)	10.25 (±6.27)	−3.5 (−4.4, −2.58)	<.001
PGA (only morphea)				
Activity	53.88 (±19.61)	18.75 (±18.08)	−35.1 (−47.1, −23.1)	<.001
Damage	55.0 (±21.04)	61.25 (±16.85)	6.25 (−4.38, 16.9)	.25
Durometer Measurements	34.55 (±15.88)	31.03 (±16.80)	−1.88 (−5.31, 1.54)	.28

*CRO*, Clinician reported outcome; *HAMIS*, Hand Mobility in Scleroderma; *LoSAI*, Localized Scleroderma Activity Index; *LoSDI*, Localized Scleroderma Damage Index; *mRSS*, modified Rodnan Skin Score; *PGA*, Physician Global Assessment; *SSc*, systemic sclerosis.

Our results suggest that UVA-1 phototherapy may improve QoL, functionality, and CROs in SSD. This supports a possible benefit for UVA-1 phototherapy in SSD for improving QoL and measures of sclerosis. It is not clear why the improvement in HAQ-DI scores was non-significant. Perhaps disability-related outcomes are more delayed than other constructs, but our limited cohort makes further inference challenging. Interpretation and generalizability are limited by small sample size, single-center cohort, continuation of concomitant treatment, and non-randomized, non-blinded study design. Including multiple diseases further reduced power for disease-specific analyses and broader generalizability. Nonetheless, these pilot data provide preliminary support for UVA-1 therapy in SSD and highlight the value of combined PRO/CRO assessment.

### Declaration of generative AI and AI-assisted technologies in the writing process

No generative AI was used in the creation of this manuscript.

## Conflicts of interest

Dr Hopkins receives consulting fees from Priovant Therapeutics and Dren Bio and is supported by a Dermatology Foundation career development award.
